# Does Urinary Bladder Shape Affect Urinary Flow Rate in Men with Lower Urinary Tract Symptoms?

**DOI:** 10.1155/2014/846856

**Published:** 2014-01-08

**Authors:** Yusuf Ziya Ateşçi, Özgü Aydoğdu, Ayhan Karaköse, Mahmut Pekedis, Ömer Karal, Utku Şentürk

**Affiliations:** ^1^Department of Urology, Izmir University School of Medicine, Uckuyular, 35200 Izmir, Turkey; ^2^Department of Mechanical Engineering, Ege University Faculty of Engineering, Bornova, 35200 Izmir, Turkey; ^3^Department of Electrics and Electronics Engineering, Dokuz Eylül University Faculty of Engineering, 35200 Izmir, Turkey

## Abstract

We aimed to investigate the role of urinary bladder shape which may potentially change with advancing age, increased waist circumference, pelvic ischemia, and loosening of the urachus on bladder emptying and UFR. We retrospectively investigated the medical records of 76 men. The patients were divided into two groups according to bladder shapes in MRI scan (cone and spheric shapes). There was a significant difference between the two groups in terms of IPSS, Qmax, Qave, and waist circumference. A positive correlation has been demonstrated between mean peak urinary flow rate measured with UFM and mean flow rate calculated using the CP. There was a significant difference between mean urinary flow rates calculated with CP of cone and sphere bladder shapes. The change in the bladder shape might be a possible factor for LUTS in men and LUTS may be improved if modifiable factors including increased waist circumference and loosening of the urachus are corrected.

## 1. Introduction

The men with lower urinary tract symptoms (LUTS) constitute a significant part of the patients referred to the urology outpatient clinics [1]. The etiology of LUTS which negatively affect the patients' quality of life especially in older ages is still not clear [2]. Previously LUTS has been advocated to be associated with bladder outlet obstruction (BOO) caused mainly by benign prostatic hyperplasia (BPH) particularly in elderly men [1, 3]. However several studies have demonstrated that LUTS and decreased urinary flow rate (UFR) may not be only associated with BOO, and the size of the prostate does not correlate with the severity of obstructive process [4–6].

Some studies have reported that age-related changes affecting the bladder smooth muscle, urothelium, vasculature, and innervation might potentially cause LUTS [2, 7–10]. Decreased perfusion in the urinary bladder has also been speculated to produce significant changes in bladder function and structure leading to the development of LUTS with advancing age [2, 11]. Obesity and increased waist circumference are other unfavorable factors which potentially affect bladder emptying.

We have previously recognized that sagittal magnetic resonance imaging (MRI) sections, which were performed when the bladder was full, demonstrated different bladder shapes changing from spherical to triangular prism (water drop shape). We speculated that the bladder shape might have a potential role in UFR reduction. We thought that there might be an association between different geometric shapes detected in sagittal sections of MRI and bladder storage and voiding pressure.

While ileum neobladder geometry is known to affect the pressure change inside the pouch, this feature is designed taking into account the geometry of the pouches. Camey pouch has small diameter and height. In previous years, on the basis of Laplace laws of physics, W pouch was preferred instead of Camey pouch. The main reason underlying this is to reduce the pressure inside the neobladder [1–6].

In the recent study, we aimed to investigate the role of urinary bladder shape which may potentially change with advancing age, increased waist circumference, pelvic ischemia, and loosening of the urachus on bladder emptying and UFR. In this study, computational fluid dynamics (CFD) based on the finite volumes solution technique was applied to determine the bladder pressure and UFR as a recent noninvasive method for the measurement of bladder pressure and UFR.

## 2. Materials and Methods

**Figure 1:**
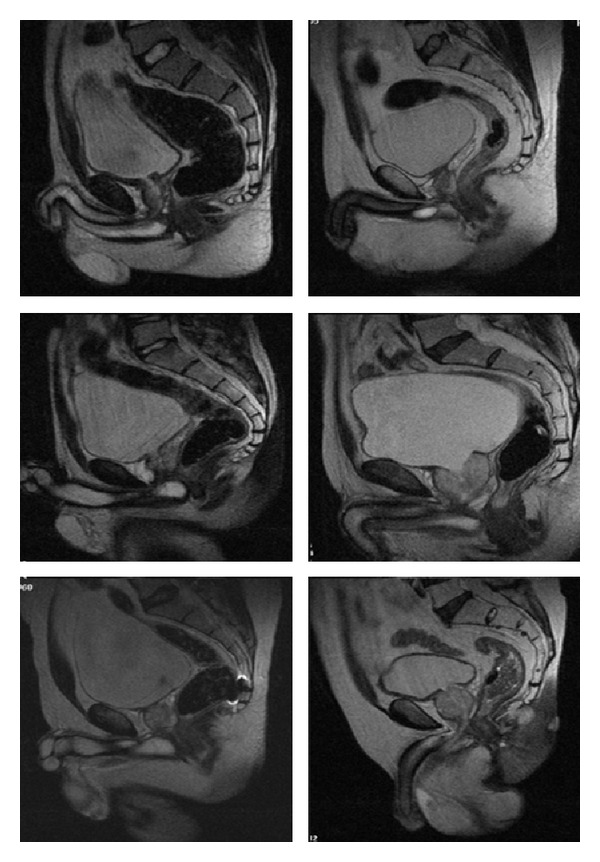
MRI images showing spheric and cone bladder shapes.

We retrospectively investigated the medical records of men who were admitted to urology outpatient clinic with lower urinary tract symptoms (LUTS) and underwent MRI for various reasons. Patients with a history of neurogenic bladder and previous prostate or urethral surgery were excluded. We realized that some patients had cone while others had spheric bladder shapes in MRI scan. Seventy-six men (mean age; 56.7 ± 10.9 years) were included in the study. The patients were divided into two groups according to bladder shapes in MRI scan (group 1, cone bladder shape, and group 2, spheric bladder shape) (Figure 1). International Prostate Symptom Score (IPSS), quality of life (QoL) score, waist circumference, and body mass index (BMI) were noted. Groups were compared in terms of maximum urinary flow rate (Qmax) and average urinary flow rate (Qave) measured with uroflowmetry (UFM), BMI, waist circumference, IPSS, and QoL score.

**Figure 2:**
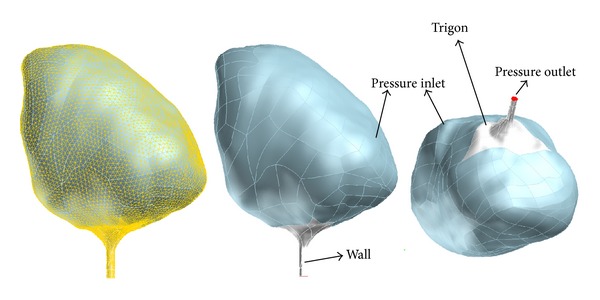
Three-dimensional (3D) urinary bladder reconstructions using MIMICS.

Three-dimensional (3D) urinary bladder reconstructions were performed using MIMICS which is a software particularly developed for medical image processing (Figure 2). All spheric bladder shapes were converted to conic and all conic shapes were converted to spheric, respectively, using the computer program. Urethral resistance, prostatic resistance, volume differences, detrusor contraction pressure, and tension and factors effecting the bladder wall thickness were equalized in both groups using the computer program to evaluate the unique effect of the bladder shape on urinary flow rate.

Initially the bladder region was identified and then transferred to the MIMICS. Following that, the bladder configuration was labeled in all sections of the MRI scans; an automatic 3D reconstruction of the soft tissues was performed creating triangulated surfaces. A smoother surface was achieved using AutoCAD software.

**Figure 3:**
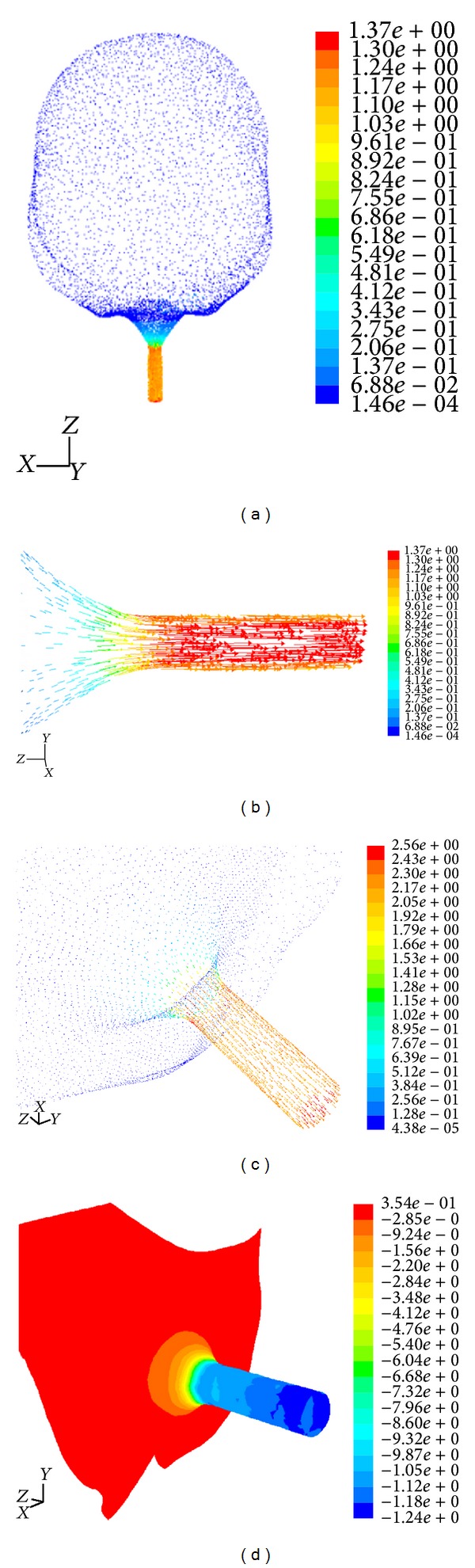
Urinary flow rate distribution in urinary bladder obtained from computational fluid dynamics (CFD) (velocity vectors colored by velocity magnitude (m/s)).

The numerical solutions were conducted in ANSYS FLUENT package to measure of the bladder pressure and UFR for reconstructed bladders (Figure 3). CFD is the process of obtaining approximate solutions for the Navier-Stokes equations of fluid motion, using numerical methods. Finite volume method was used to solve these equations and provide the urinary flow rate and pressure fields in the bladder.

We investigated if there is a correlation between UFRs measured with uroflowmetry and CFD. Mean urinary flow rates measured with CFD were compared between cone and sphere bladder shapes. Statistical analyses were performed with SPSS 15.0, and a value of *P* < 0.05 was considered as statistically significant.

## 3. Results

Group 1 included 41 patients with conic bladder shape and group 2 included 35 patients with spheric bladder shape. Mean age of the patients was 51.3 ± 4.3 and 62.1 ± 2.9 in groups 1 and 2, respectively, (*P* < 0.05). No significant difference was noted between the two groups in terms of QoL score and BMI. There was a significant difference between the two groups in terms of IPSS, Qmax, Qave, see and waist circumference (Table 1).

**Figure 4:**
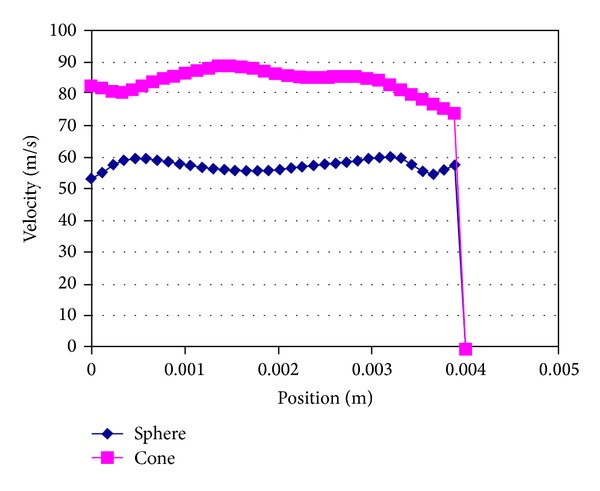
Urinary flow rate values obtained from CFD.

A positive correlation (correlation coefficient = 0.634, *P* = 0.02) has been demonstrated between mean peak urinary flow rate measured with uroflowmetry and mean flow rate calculated using the computer program (21.3 ± 3.9 mL/s and 22.7 ± 1.7 mL/s, resp.). The mean bladder pressure was calculated as 16.8 ± 6.4 cmH_2_O with computer program. There was a significant difference between the bladder with cone shape and the bladder with sphere shape in terms of mean flow rates calculated in CFD (78.01 and 46.22 m/s, resp.) (*P* = 0.003) (Figure 4).

## 4. Discussion

Previous studies showed that ageing is associated with significant deterioration in physiological functions of organ systems including urinary system [2, 12]. The incidence of LUTS is increasing with advancing age and unfavorable effects of LUTS on daily life may potentially be as influential as various life-threatening concomitant diseases [2, 13]. Although LUTS are generally attributed to bladder outlet obstruction mostly caused by enlarged prostate, there is no consensus on the etiology of LUTS [2, 6].

In a previous study, 464 men were investigated using transrectal ultrasound scan (TRUS) evaluation of prostatic volume, measurement of urinary flow rates, and the American Urological Association (AUA) symptom score [14]. The authors reported only a very weak relationship between prostate size and the symptom score. Various studies have reported that the size of the prostate does not correlate with the degree of infravesical obstruction [4, 5]. Several studies have demonstrated changes in bladder function affecting both genders with advancing age [2, 12, 14]. Aforementioned studies advocated that the pathophysiological basis of LUTS in elderly patients might potentially involve age-associated changes affecting the smooth muscle, urothelium, vasculature, and innervation of the bladder.

Previously it has been speculated that age-related impairment of blood supply to the prostate might have a key role in the pathophysiology of LUTS, since lower urinary bladder perfusion has been demonstrated in elderly patients with LUTS when compared to younger patients [2, 11]. Other parameters including transitional zone volume (TZV), TZI, IPP, prostate configuration, and prostatic urethral angulation (PUA) have been investigated as possible age-related factors affecting bladder emptying [1, 15–17]. In a previous study the authors reported a significant correlation between TZI and peak urinary flow rate (Qmax) measured with uroflowmetry [8]. In contrast, in another study TPV, TZV, and TZI were found to be weakly related to Qmax in patients with clinical BPH [17]. In a recent study, Bang et al. investigated the effect of the PUA on urinary symptoms and Qmax in patients with BPH/LUTS in a clinical setting [1]. The authors have demonstrated an inverse relationship between PUA and urinary flow rate. The mechanisms by which ageing leads to functional changes in the urinary bladder have not been well understood in spite of several previous studies which aimed to clarify the pathophysiology of LUTS.

In the recent study, we aimed to evaluate the possible role of urinary bladder shape in men with LUTS. Mean age was significantly higher in patients with spheric bladder shape when compared to conic. This finding can be interpreted as urinary bladder shape probably changes from cone to spheric with advancing age, thus affecting the urinary flow rate. We used the computational fluid dynamics based on finite volumes solution technique which were previously used to evaluate prostatic obstruction and dynamic analyses of bladder-urethra system [18, 19]. Following 3D urinary bladder reconstructions of MRI images as cone and sphere with MIMICS software, CFD was applied to measure the urinary flow rate for reconstructed bladders. Mean urinary flow rates at the proximal urethra measured with computer program were compared between cone and sphere bladder shapes. Potential correlation between urinary flow rates measured with uroflowmetry and CFD was also evaluated. A positive correlation has been demonstrated between mean peak urinary flow rate measured with uroflowmetry and mean flow rate calculated with CFD software. The mean urinary flow rates were significantly higher in cone bladder shape when compared to the sphere.

Our findings demonstrated a significantly higher mean waist circumference in patients with spheric when compared to cone bladder shape detected in MRI scan. Although there was a statistical difference between the two groups in terms of waist circumference, no significant difference was noted in terms of BMI. We thought that the main reason for the change in bladder shape from cone to sphere with advancing age is increased waist circumference instead of increased BMI.

Previously some investigators have endeavored to explain the pathophysiology of LUTS in men, and various factors related to the urinary bladder, urethra, and prostate have been advocated to be associated with LUTS. To the best of our knowledge, the recent study represents the first trial investigating the unique potential role of bladder shape on LUTS in men. Bladder shape may potentially change from cone to sphere due to advancing age, increased waist circumference, and thus loosening of the urachus. Our findings suggest that LUTS may be improved if modifiable factors including increased waist circumference and loosening of the urachus, are corrected. The change in the bladder shape might be a causal factor for LUTS in men. The use of computational fluid dynamic solution procedure with MRI data can potentially replace invasive diagnostic tools in the management of LUTS in men. In addition, bladder shape detected in preoperative MRI scan could potentially guide the clinicians to evaluate which patients would benefit more from a surgery for infravesical obstruction.

The present study includes relatively elderly patients with LUTS. It would probably be more significant if we could evaluate younger patients without LUTS to make an objective conclusion if the bladder shape has a potential role in LUTS. Therefore further prospective and randomized studies including younger patients without LUTS are needed to support our findings. Even though the recent study has some limitations, as the authors we expect our findings to provide new perspectives for the pathogenesis and management of LUTS.

## 5. Conclusion

The pathophysiology of LUTS is possibly associated with several factors. The present study showed that the change in the bladder shape might be a possible factor for LUTS in men and LUTS may be improved if modifiable factors including increased waist circumference and loosening of the urachus are corrected.

## Figures and Tables

**Table 1:** Patients' characteristics in groups 1 and 2.

	Cone bladder shape (n = 41)	Spheric bladder shape (n = 35)	*P*
Mean age (years)	51.3 ± 4.3	62.1 ± 2.9	<0.05
IPSS	15.4 ± 2.1	20.2 ± 2.6	<0.05
QoL score	4.7 ± 0.7	4.5 ± 1.2	>0.05
Qmax (mL/s)	19.3 ± 2.1	12.3 ± 1.5	<0.05
Qave (mL/s)	8.2 ± 1.1	4.7 ± 0.9	<0.05
BMI	26.2 ± 1.9	27.1 ± 1.6	>0.05
Waist circumference (cm)	92.3 ± 6.1	103.1 ± 4.9	<0.05

IPSS: International Prostate Symptom Score, QoL: quality of life score, Qmax: maximum urinary flow rate measured with uroflowmetry, Qave: mean urinary flow rate measured with uroflowmetry, and BMI: body mass index.
