# Erratum to “Does Urinary Bladder Shape Affect Urinary Flow Rate in Men with Lower Urinary Tract Symptoms?”

**DOI:** 10.1155/2014/462623

**Published:** 2014-03-18

**Authors:** Yusuf Ziya Ateşçi, Özgü Aydoğdu, Ayhan Karaköse, Mahmut Pekedis, Ömer Karal, Utku Şentürk, Murat Çınar

**Affiliations:** ^1^Department of Urology, Faculty of Medicine, Izmir University, Karsiyaka, 35200 Izmir, Turkey; ^2^Department of Mechanical Engineering, Faculty of Engineering, Ege University, Bornova, 35040 Izmir, Turkey; ^3^Department of Electrics and Electronics Engineering, Faculty of Engineering and Natural Sciences, Yıldırım Beyazıt University, 06050 Ankara, Turkey; ^4^Department of Biomedical Engineering, Faculty of Engineering and Architecture, Izmir Katip Celebi University, 35620 Izmir, Turkey

In the paper titled “Does Urinary Bladder Shape Affect Urinary Flow Rate in Men with Lower Urinary Tract Symptoms?” we should add an author who has contributed to our study for the images: Murat Çınar, Department of Biomedical Engineering, Faculty of Engineering and Architecture, Izmir Katip Celebi University, 35620 Izmir, Turkey.

In addition we should here correct the affiliation of one of the authors named Ömer Karal and the postal code in the affiliation of the author Mahmut Pekedis.

